# Comparison of local infiltration analgesia and sciatic nerve block for pain control after total knee arthroplasty: a systematic review and meta-analysis

**DOI:** 10.1186/s13018-017-0586-z

**Published:** 2017-06-07

**Authors:** Li-ping Ma, Ying-mei Qi, Dong-xu Zhao

**Affiliations:** 1 China-Japan Union Hospital of Jilin University, Changchun, People’s Republic of China; 2 Department of Orthopedics, China-Japan Union Hospital of Jilin University, 126 Xiantai Street, Changchun, Jilin People’s Republic of China

**Keywords:** Sciatic nerve block, Local infiltration analgesia, Total knee arthroplasty, Pain control, Meta-analysis

## Abstract

**Background:**

This meta-analysis aimed to perform a meta-analysis to evaluate the efficiency and safety between local infiltration analgesia (LIA) and sciatic nerve block (SNB) when combined with femoral nerve block (FNB) after total knee arthroplasty (TKA).

**Methods:**

A systematic search was performed in MEDLINE (1966-2017.04), PubMed (1966-2017.04), Embase (1980-2017.04), ScienceDirect (1985-2017.04), and the Cochrane Library. Only high-quality studies were selected. Meta-analysis was performed using Stata 11.0 software.

**Results:**

Four randomized controlled trials (RCTs) and two non-randomized controlled trials (non-RCTs), including 273 patients met the inclusion criteria. The present meta-analysis indicated that there were significant differences between groups in terms of visual analogue scale (VAS) score at 12 h (SMD = −0.303, 95% CI −0.543 to −0.064, *P* = 0.013), VAS score at 24 h (SMD = −0.395, 95% CI −0.636 to −0.154, *P* = 0.001), morphine equivalent consumption at 24 h (SMD = −0.395, 95% CI −0.636 to −0.154, *P* = 0.001), and incidence of nausea (RD = 0.233, 95% CI 0.107 to 0.360, *P* = 0.000) and vomiting (RD = 0.131, 95% CI 0.025 to 0.237, *P* = 0.015).

**Conclusion:**

FNB-combined SNB provides superior pain relief and less morphine consumption within the first 24 h compared FNB-combined LIA in total knee arthroplasty. In addition, there were fewer side effects associated with SNB. Because the sample size and the number of included studies were limited, a multicenter RCT is needed to identify the effects of the two kinds of methods and further work must include range of motion analyses and functional test.

## Background

Total knee arthroplasty (TKA) is a common procedure for improving mobility and quality of life in patients with osteoarthritis or rheumatoid arthritis. However, it is reported that 30–60% of patients suffer moderate to severe postoperative pain [1]. Adequate and effective pain relief is requested, mainly to improve patient satisfaction, to expedite mobilization and rehabilitation, to decrease the duration of hospital stay, and consequently to lower the risk of deep vein thrombosis or nosocomial infections [2–4]. Femoral nerve block (FNB) could provide effective analgesia and is a well-accepted method for regional anesthesia following TKA [5, 6]; however, some patients still experienced significant postoperative pain. Compared with FNB, local infiltration anesthesia (LIA) is an alternative and cost-effective anesthetic technique which has been promoted for a few decades and shows excellent outcome for pain relief after TKA [7, 8]. Previous studies have reported that LIA was comparable to epidural anesthesia and FNB for analgesic effect in total joint arthroplasty. LIA is considered as a promising method with few side effects and prospective of early mobilization without weakness of quadriceps muscle strength [9, 10]. Therefore, LIA is a major choice for supplementing FNB after TKA. However, fundamental research has shown that knee joint is also innervated by sciatic nerves; thus, FNB combined sciatic nerves block (SNB) has become growing practice to provide improved pain relief.

However, there is no consensus regarding which anesthesia method is preferable to relieve pain as an adjunct to FNB. Thus, a meta-analysis of randomized controlled trials (RCTs) was conducted to compare the efficacy and safety of pain control with SNB versus LIA when combined with FNB after TKA.

## Methods

### Search strategy

**Fig. 1:**
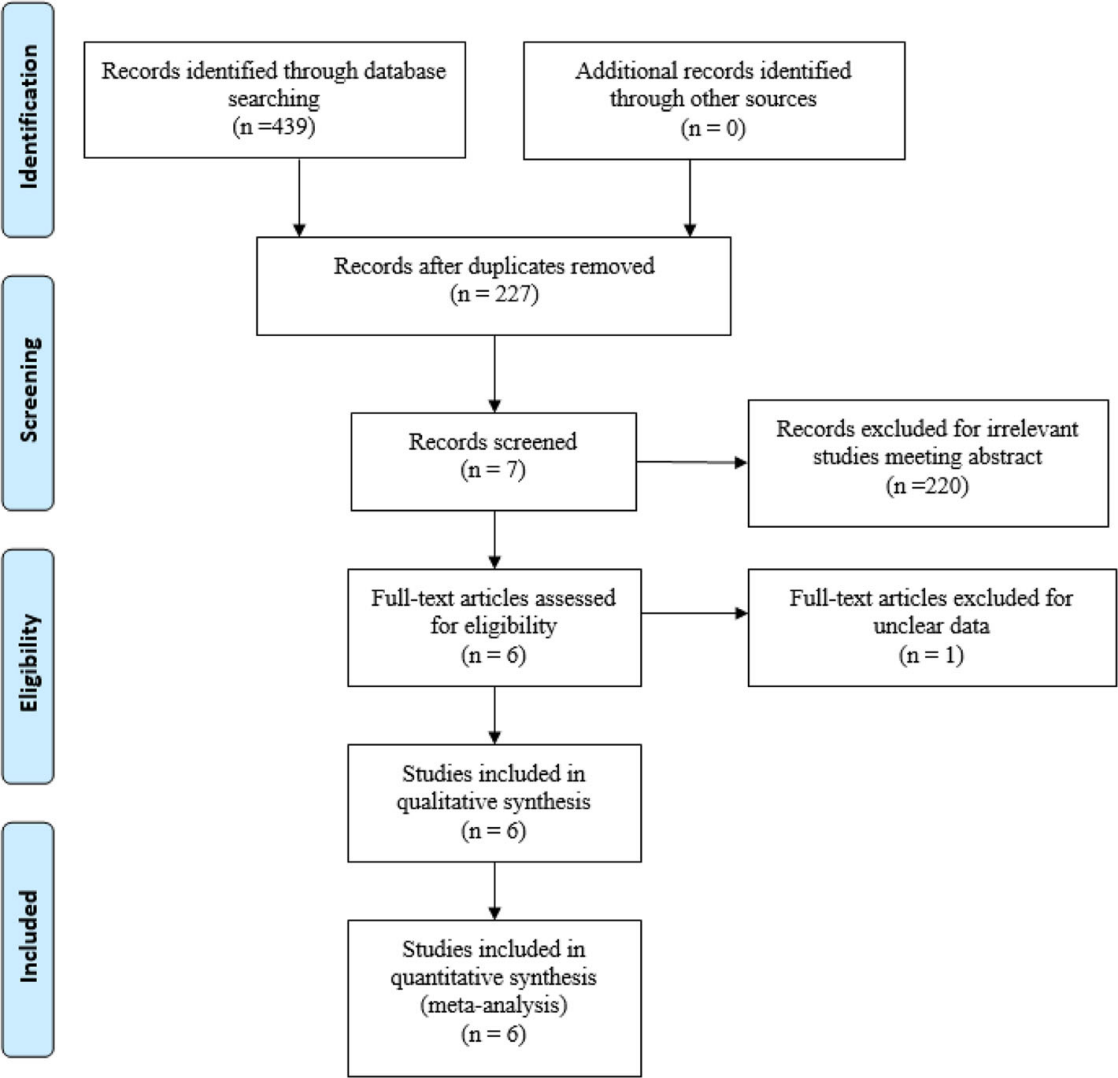
Search results and the selection procedure

Potentially relevant studies were identified from electronic databases including MEDLINE (1966-2017.4), PubMed (1966-2017.4), Embase (1980-2017.4), ScienceDirect (1985-2017.4), and the Cochrane Library. The following keywords were used in combination with the Boolean operators AND or OR: “total knee replacement OR arthroplasty,” “femoral nerve block,” “sciatic nerves block,” “local infiltration anesthesia,” and “pain control.” The bibliographies of the retrieved trials and other relevant publications were cross-referenced to identify additional articles. We placed no restrictions on the publication language. The search process was performed as presented in Fig. 1.

### Inclusion and exclusion criteria

Studies were considered eligible if they met the following criteria: (1) Published clinical randomized control trails (RCTs) and non-RCTs; (2) Patients undergoing TKA, experiment group received SNB-combined FNB for pain control and control group received LIA-combined FNB; (3) Reported surgical outcomes, including visual analogue scale (VAS) scores, morphine consumption, length of stay, and postoperative adverse effects including the risk of nausea and vomiting. Studies would be excluded from present meta-analysis for incomplete data, case reports, conference abstract, or review articles.

### Selection criteria

Two reviewers independently review the abstract of the potential studies. After an initial decision, full text of the studies that potentially met the inclusion criteria were reviewed and final decision was made. A senior reviewer is consulted in case of disagreement.

### Date extraction

Two reviewers independently extracted the relevant data from the included studies. Details of incomplete data of included articles are received by consulting the corresponding author. Following data was extracted: first author names, published year, study design, comparable baseline, anesthesia methods, and dosage and type of anesthetic drug. Outcome parameters included VAS scores at different periods, the cumulative morphine consumption, length of stay, and postoperative adverse effects. Other relevant data was also extracted from individual studies.

### Quality assessment

Quality assessment of included studies was performed by two reviewers independently. Modified Jadad score (7-point scale) which was based on Cochrane Handbook for Systematic Reviews of Interventions is used for assessment of RCTs. Studies which scores greater than four points was considered high quality. We conducted “risk of bias” table including the following key points: random sequence generation, allocation concealment, blinding, incomplete outcome data, free of selective reporting, and other bias. The Methodological Index for Non-Randomized Studies (MINORS) scale was used to assess non-RCTs with scores ranging from 0 to 24. A consensus is reached through a discussion.

### Data analysis and statistical methods

All calculation was carried out by Stata 11.0 (The Cochrane Collaboration, Oxford, UK). Statistical heterogeneity was assessed based on the value of *P* and *I* ^2^ using standard chi-square test. When *I* ^2^ > 50%, *P* < 0.1 was considered to be significant heterogeneity; random-effect model was performed for meta-analysis. Otherwise, fixed-effect model was used. If possible, sensibility analysis is conducted to explore the origins of heterogeneity. The results of dichotomous outcomes were expressed as risk difference (RD) with a 95% confidence intervals (CIs). For continuous various outcomes, mean difference (MD) and standard mean difference (SMD) with a 95% confidence intervals (CIs) were applied for assessment.

## Results

### Search result

A total of 439 studies were preliminarily reviewed. By reading the title and abstracts, 433 reports were excluded from current meta-analysis followed inclusion criteria. No gray reference was obtained. Finally, four RCTs [11–14] and two non-RCTs [15, 16] which had been published between 2014 and 2016 were enrolled in present meta-analysis and includes 136 participates in the SNB groups and 137 patients in the LIA groups.

### Risk of bias assessment

**Fig. 2:**
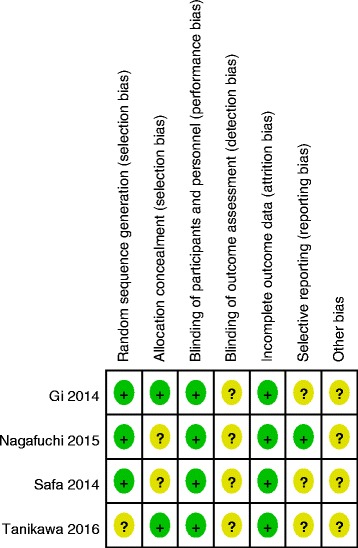
Methodological quality of the randomized controlled trials

**Fig. 3:**
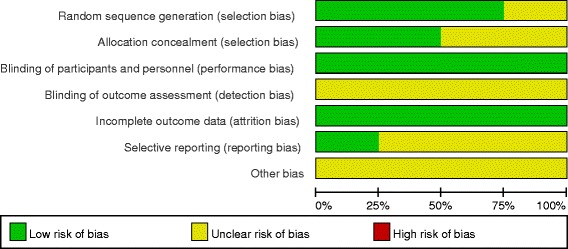
Risk of bias

Demographic characteristics, the details about the included studies are summarized in Table 1. Modified Jadad score which was based on Cochrane Handbook for Systematic Reviews of Interventions is used for assessment of RCTs (Fig. 2). All RCTs [11–14] provide clear inclusion and exclusion criteria and suggest a methodology of randomization, two [12–14] of which described that randomization algorithm was generated from computer. Two of them [11, 13] stated allocation concealment was achieved by sealed envelope. Double blinding was provided in all RCTs. None of them had stated assessors were blinded. Each risk of bias item is presented as the percentage across all included studies, which indicates the proportion of different levels of risk of bias for each item (Fig. 3). All RCTs demonstrated complete outcome data. The MINORS scale was used to assess non-RCTs by assigning scores ranging from 0 to 24 (Table 2).

**Table 1:** Trials characteristics

Studies	Reference type	Cases	Mean age	Female patient	Anesthesia	Drug dose of FNB	Drug dose of SNB	Drug dose of LIA	Concomitant Pain	Follow-up
		(SNB/LIA)	(SNB/LIA)	(SNB/LIA)						
Tanikawa 2014 [11]	RCT	23/23	72/71	19/20	General anesthesia	20 ml of 0.375% ropivacaine	20 ml of 0.375% ropivacaine	200 mg of ropivacaine and 0.5 ml of adrenaline	IV ketorolac 30 mg, ketoprofen 100 mg, or diclofenac 75 mg	3 months
Gi 2014 [13]	RCT	24/25	78/77	21/24	General anesthesia	20 ml 0.375% ropivacaine	20 ml 0.375% ropivacaine	60 ml 0.5% ropivacaine with 0.3 mg epinephrine	400 mg celecoxib, 20 mg oxycontin, and a 6 mg scopolamine patch topically	1 month
Safa 2014 [12]	RCT	33/32	61/61	18/15	Spinal anesthesia	20 mL of 0.5% ropivacaine	20 mL of 0.5% ropivacaine	50 mL of 0.2% ropivacaine	Celecoxib 200 mg, gabapentin 200 mg and acetaminophen 1 g	1.5–3 months
Nagafuchi 2015 [14]	RCT	17/16	72/73	15/13	General anesthesia	20 mL of 0.375% ropivacaine	20 ml of 0.375% ropivacaine	100 mL of 0.2% ropivacaine	Celecoxib 200 mg, gabapentin 200 mg and acetaminophen 1 g	1 months
Cip 2016 [15]	Non-RCT	16/18	73.4/71.8	12/11	Spinal or general anesthesia	0.2% ropivacaine (4 ml/h)	20 ml ropivacaine 0.2%	0.33% ropivacaine (5 mL/h)	Celecoxib and Oxycodone	NS
Aikawa 2016 [16]	Non-RCT	23/23	72/71	19/20	general anesthesia	20 ml 0.375% ropivacaine	20 ml 0.375% ropivacaine	20 mL of 0.375% levobupivacaine	NS	6 months

*SNB* sciatic nerve block, LIA local infiltration of analgesia, *IV* intravenous, *NS* not stated

**Table 2:** Methodological quality of the non-randomized controlled trials

Quality assessment for non-randomized trials	Cip 2016 [15]	Aikawa 2016 [16]
A clearly stated aim	2	2
Inclusion of consecutive patients	2	2
Prospective data collection	2	2
Endpoints appropriate to the aim of the study	2	2
Unbiased assessment of the study endpoint	0	0
A follow-up period appropriate to the aims of study	2	2
Less than 5% loss to follow-up	2	2
Prospective calculation of the sample size	0	2
An adequate control group	2	2
Contemporary groups	0	1
Baseline equivalence of groups	2	2
Adequate statistical analyses	2	2
Total score	18	21

### Study characteristics

The sample size of the included studies ranged from 33 to 65. All of them compared efficiency and safety between SNB and LIA as a supplement for pain control in TKA. Experimental groups received SNB-combined FNB, while control groups received LIA-combined FNB. There is variation dosage and type of anesthetic drugs in included studies. Four studies [11, 13–15] applied general anesthesia and one [12] applied spinal anesthesia. Five [11–15] studies reported that surgical procedure was performed by same surgeons. All studies reported that postoperative medication was used for concomitant pain management. All of them suggest the outcomes for at least 95% of the patients. The follow-up period ranged from 1 to 3 months.

### Outcomes for meta-analysis

#### VAS scores at 12 h

**Fig. 4:**
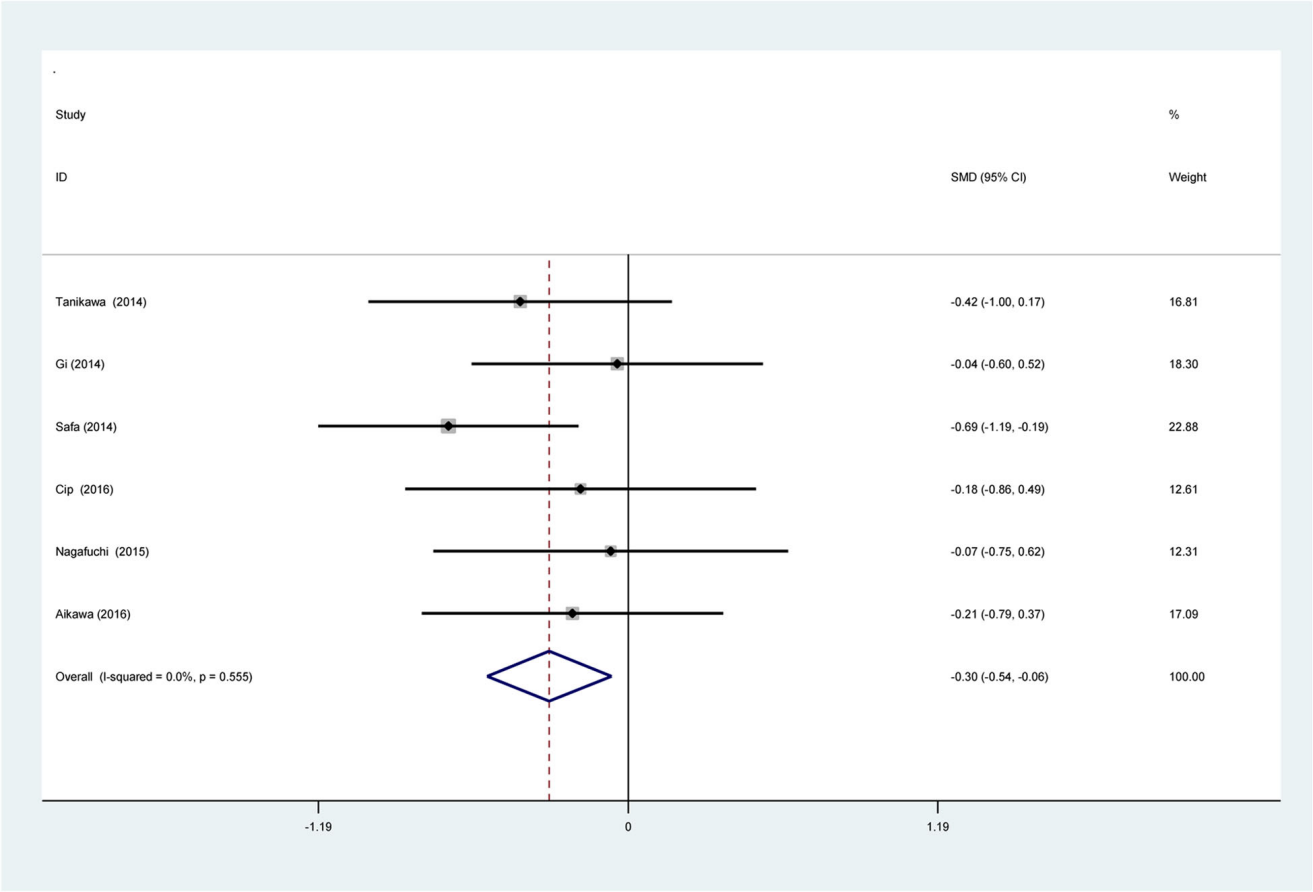
Forest plot diagram showing VAS scores at 12 h following TKA

Six studies [11–16] reported VAS scores at 12 h following TKA. There was no significant heterogeneity (*χ*2 = 3.96, df = 5, *I* ^2^ = 0%, *P* = 0.555); therefore, a fixed-effects model was used. The result of meta-analysis showed that there was significant difference between the SNB and LIA groups regarding the VAS scores at 12 h (SMD = −0.303, 95% CI −0.543 to −0.064, *P* = 0.013; Fig. 4).

#### VAS scores at 24 h

**Fig. 5:**
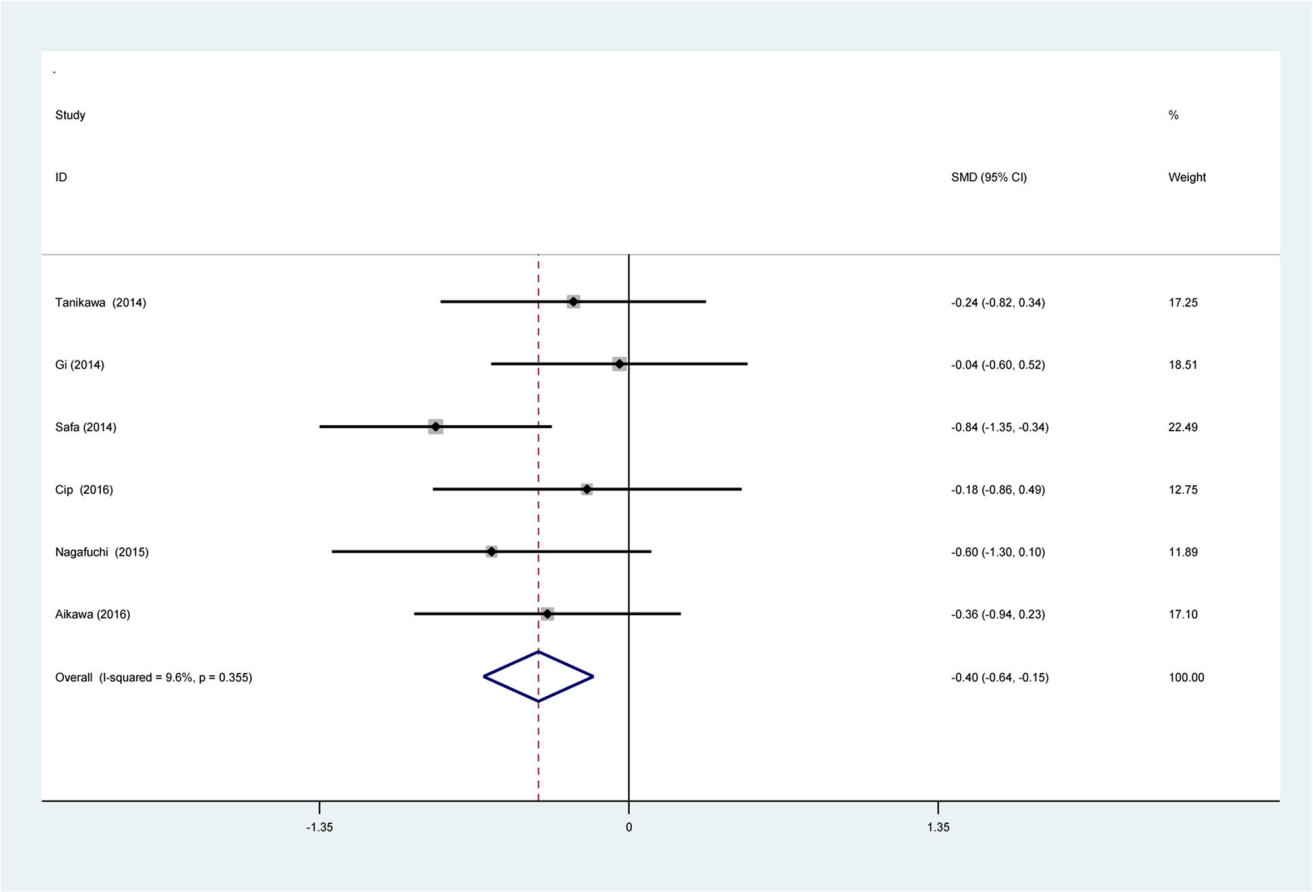
Forest plot diagram showing VAS scores at 24 h following TKA

Six studies [11–16] reported VAS scores at 24 h following TKA. No statistical heterogeneity was observed in present meta-analysis (*χ*2 = 5.53, df = 5, *I* ^2^ = 9.6%, *P* = 0.355); therefore, a fixed-effects model was applied. We found that there was significant difference between the SNB and LIA groups regarding the VAS scores at 24 h (SMD = −0.395, 95% CI −0.636 to −0.154, *P* = 0.001; Fig. 5).

#### VAS scores at 48 h

**Fig. 6:**
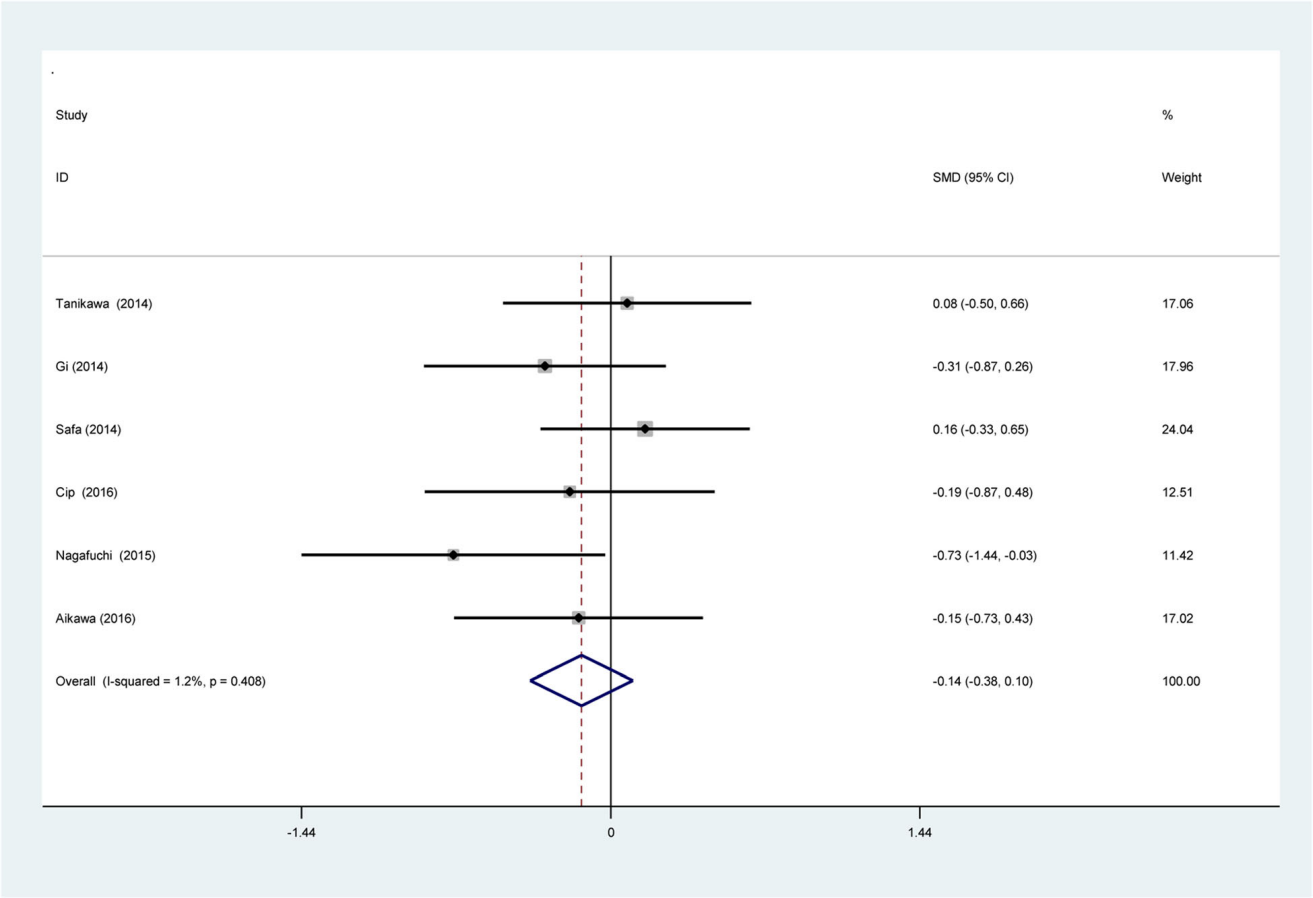
Forest plot diagram showing VAS scores at 48 h following TKA

Six reports [11–16] showed VAS scores at 48 h following TKA. There was no significant heterogeneity and a fixed-effects model was performed (*χ*2 = 5.06, df = 5, *I* ^2^ = 1.2%, *P* = 0.408). Current meta-analysis indicated that no significant difference was found in terms of VAS scores at 48 h (SMD = −0.137, 95% CI −0.375 to 0.102, *P* = 0.262; Fig. 6).

#### Morphine consumption at 24 h

**Fig. 7:**
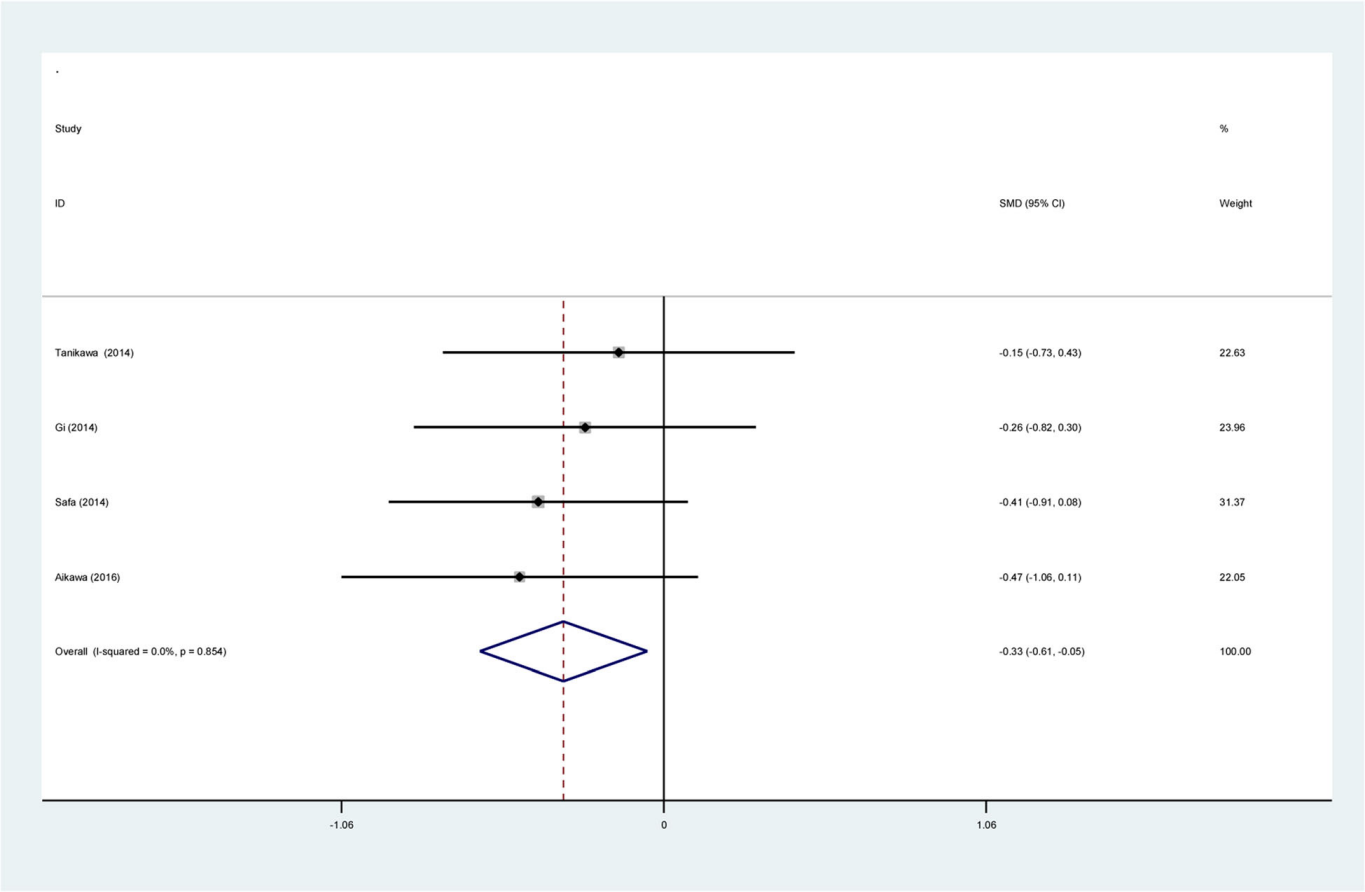
Forest plot diagram showing morphine consumption at 24 h following TKA

Morphine consumption at postoperative 24 h was presented in four studies [11–13, 16] following TKA. There was no significant heterogeneity (*χ*2 = 0.78, df = 3, *I* ^2^ = 0%, *P* = 0.854) and a fixed-effects model was used. The present meta-analysis showed that there was significant difference between the SNB and LIA groups in terms of morphine consumption at postoperative 24 h (SMD = −0.330, 95% CI −0.606 to −0.055, *P* = 0.019; Fig. 7).

#### Morphine consumption at 48 h

**Fig. 8:**
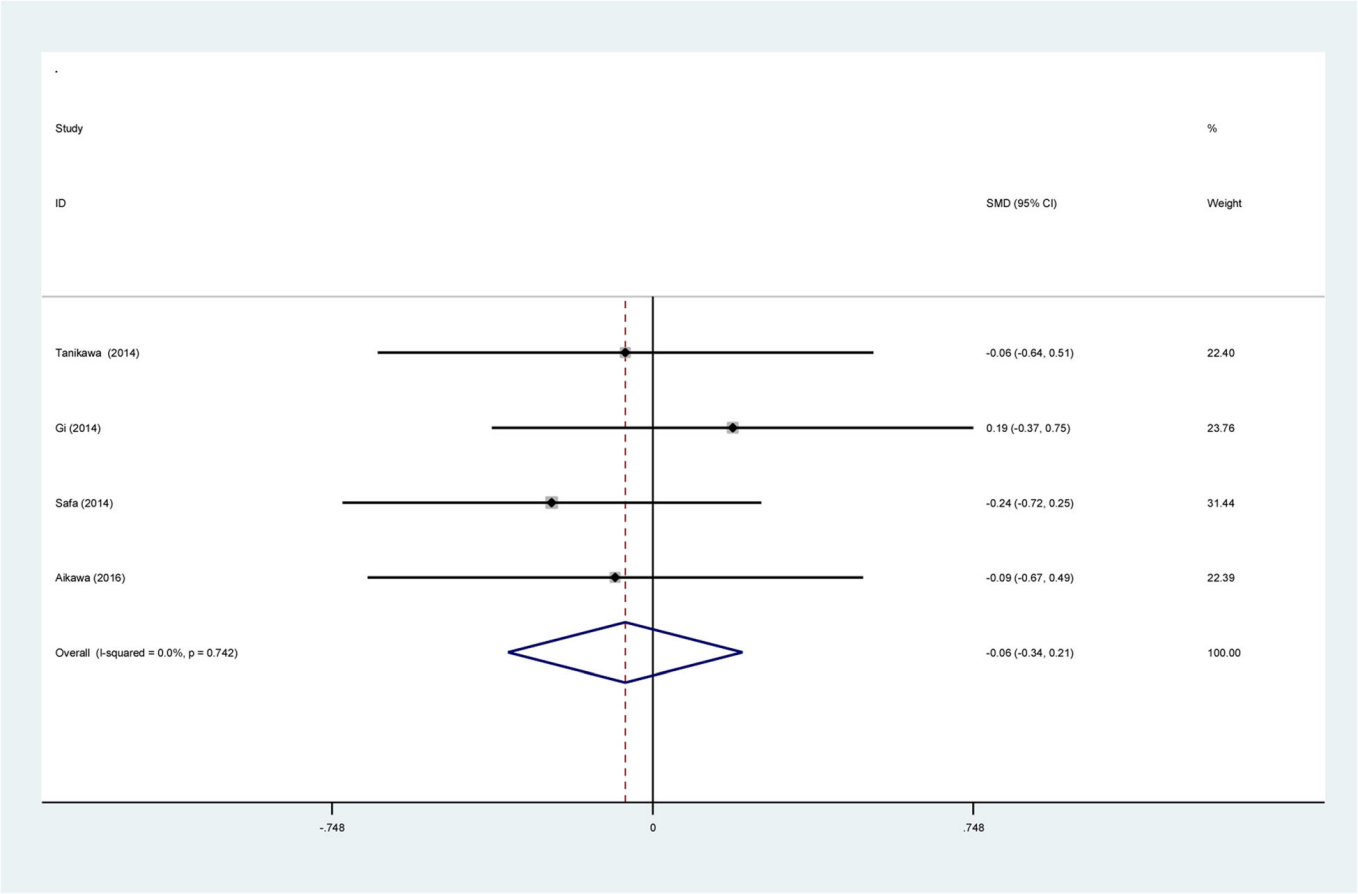
Forest plot diagram showing morphine consumption at 48 h following TKA

Four studies [11–13, 16] provided morphine consumption at postoperative 48 h following TKA. No significant heterogeneity was found (*χ*2 = 1.25, df = 3, *I* ^2^ = 0%, *P* = 0.742); therefore, a fixed-effects model was used. Meta-analysis revealed that there was no significant difference between the SNB and LIA groups in terms of morphine consumption at postoperative 48 h (SMD = −0.063, 95% CI −0.337 to 0.210, *P* = 0.649; Fig. 8).

#### Length of hospital stay (LOS)

**Fig. 9:**
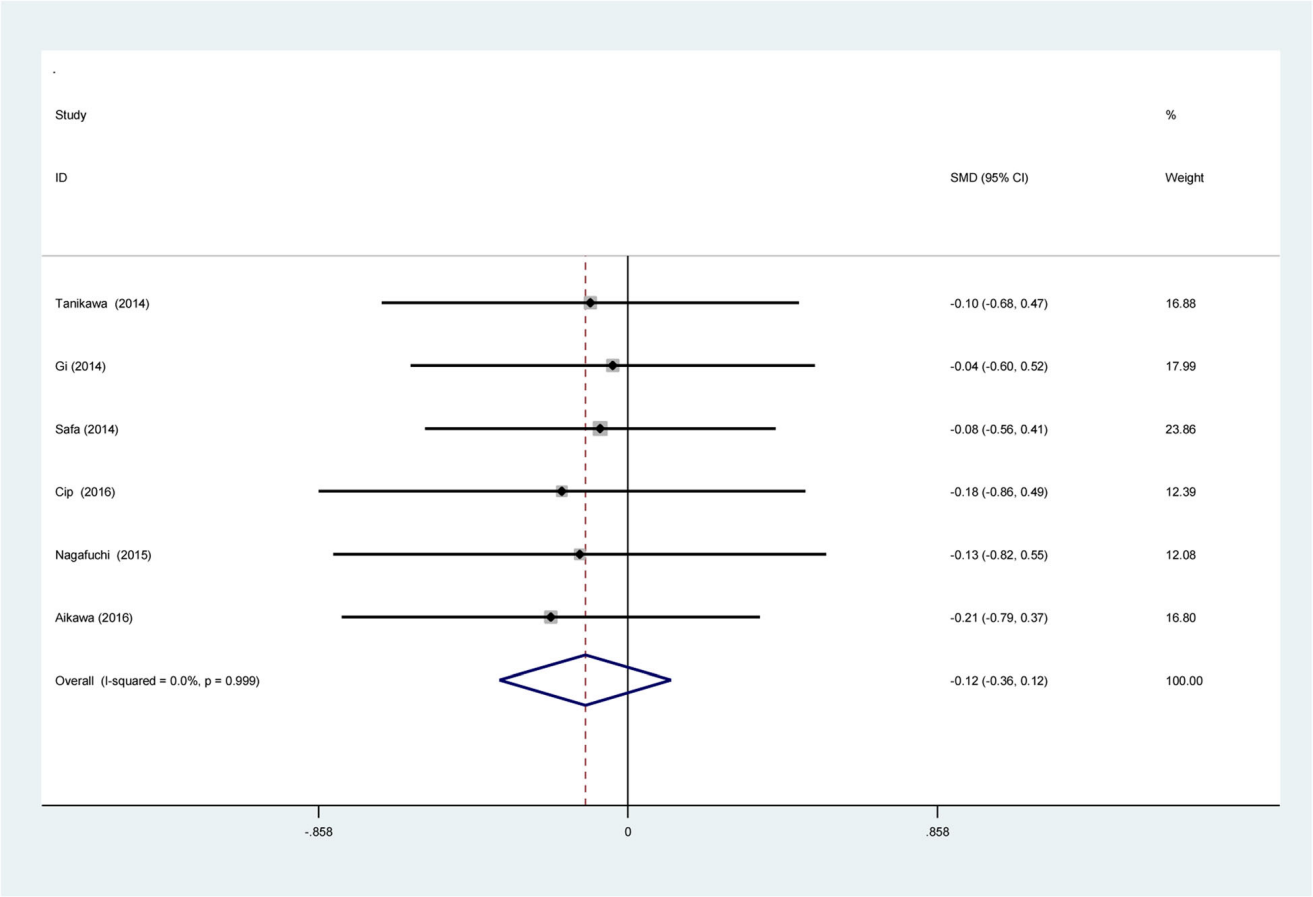
Forest plot diagram showing length of stay following TKA

Six studies [11–16] reported the length of hospital stay between groups. No significant heterogeneity was identified in the pooled results; therefore, a fixed-effects model was used (*χ*2 = 0.24, df = 5, *I* ^2^ = 0%, *P* = 0.999). There was no significant difference between the two groups in LOS (SMD = −0.118, 95% CI −0.356 to 0.120, *P* = 0.330; Fig. 9).

#### The occurrence of nausea

**Fig. 10:**
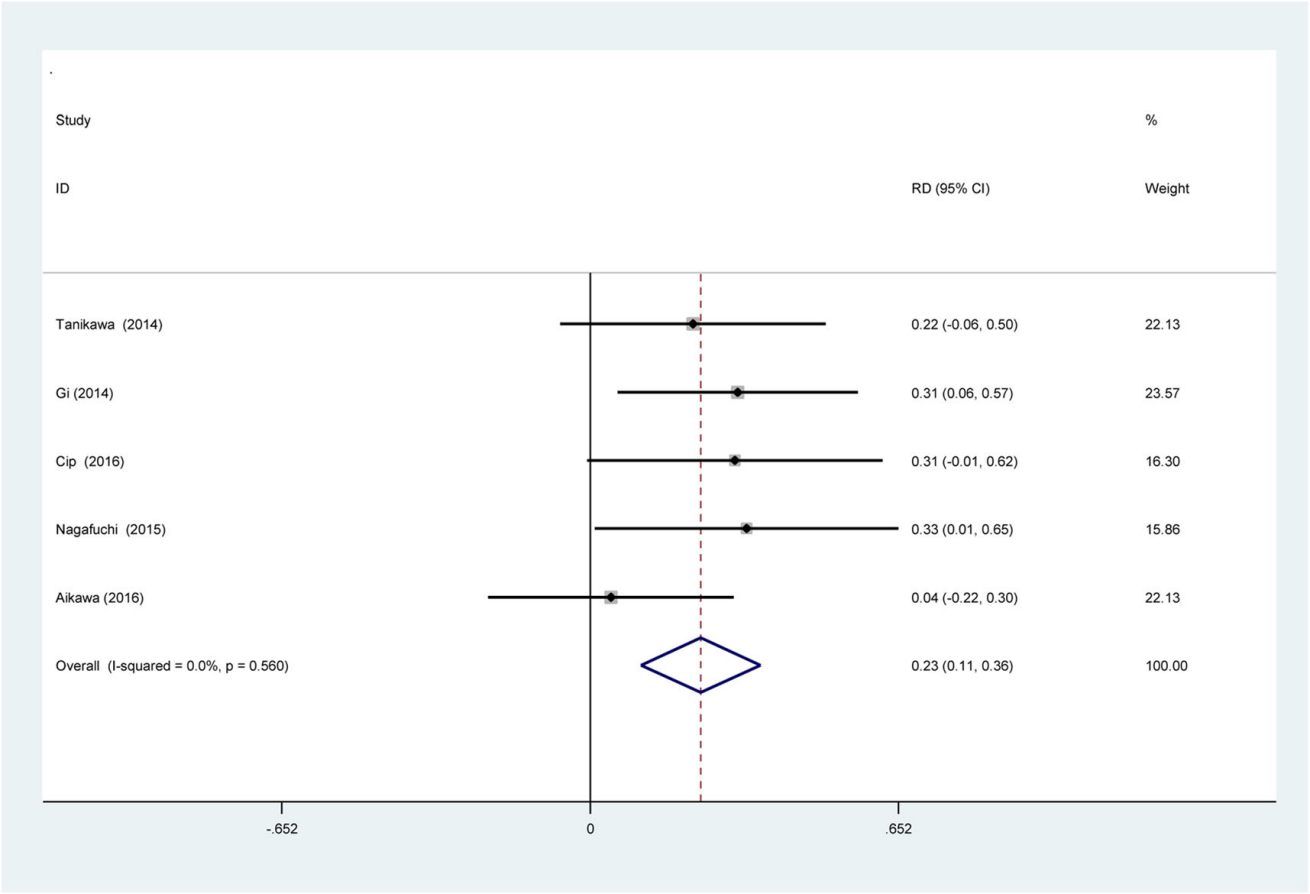
Forest plot diagram showing incidence of nausea following TKA

The occurrence of nausea was reported in five studies [11, 13–16]. No significant heterogeneity among these studies was found; therefore, a fixed-effects model was used (*χ*2 = 2.99, df = 4, *I* ^2^ = 0%, *P* = 0.560). There was significant difference between the two groups in the incidence of nausea (RD = 0.233, 95% CI 0.107 to 0.360, *P* = 0.000; Fig. 10).

#### The occurrence of vomiting

**Fig. 11:**
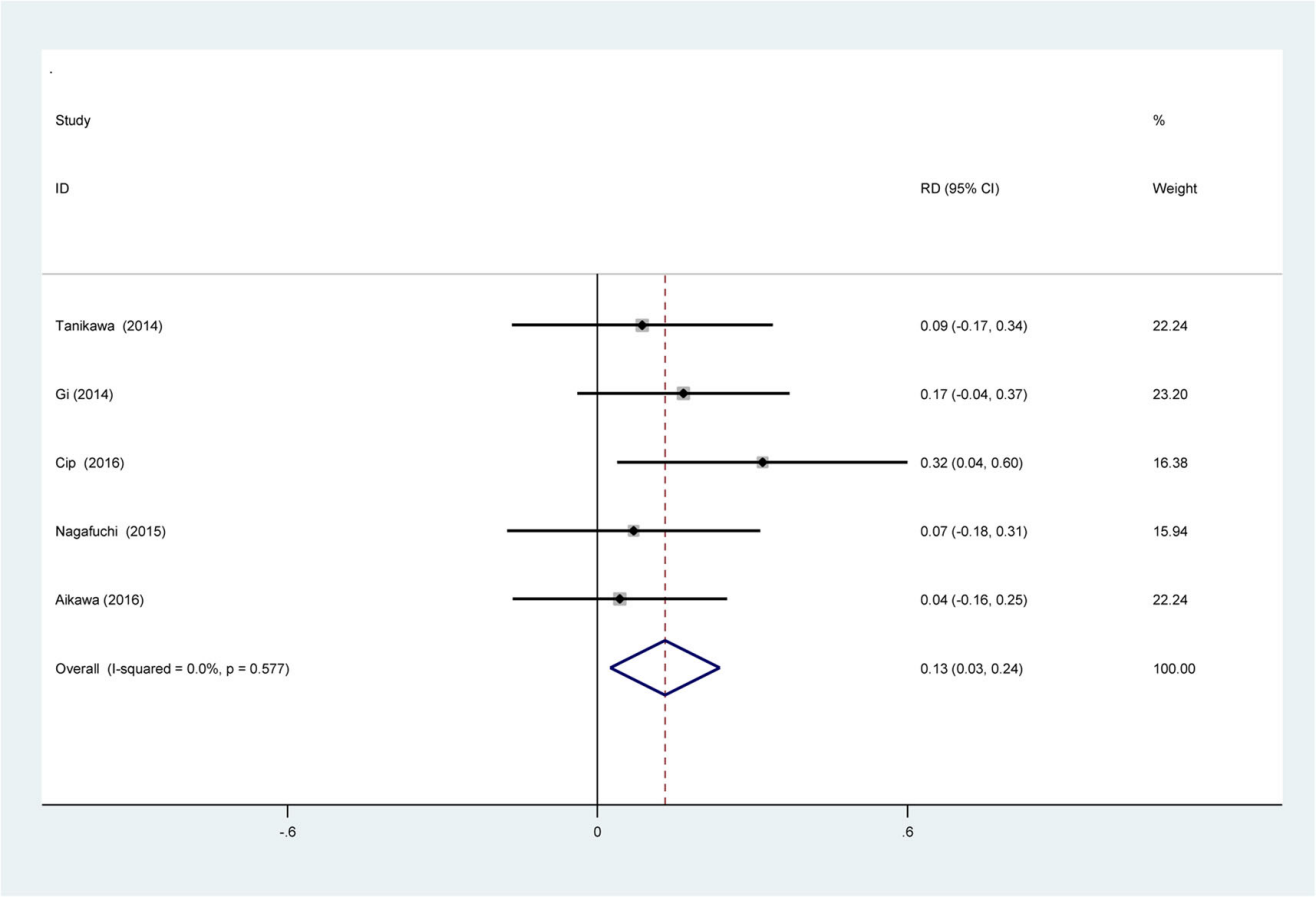
Forest plot diagram showing incidence of vomiting following TKA

Five studies [11, 13–16] reported the incidence of vomiting. We found no statistical heterogeneity and a fixed-effects model was applied (*χ*2 = 2.89, df = 4, *I* ^2^ = 0%, *P* = 0.577). Present meta-analysis showed significant difference regarding the frequency of vomiting between groups (RD = 0.131, 95% CI 0.025 to 0.237, *P* = 0.015; Fig. 11).

## Discussion

This is the first systematic review and meta-analysis to compare the efficiency and safety of combined femoral and SNB versus combined femoral with LIA for pain control in TKA. The most important finding of the present meta-analysis was that SNB-combined FNB was associated with significantly decreased pain scores at 12- to 48-h point and reduced opioids consumption at 24-h point following TKA. In addition, there was a decreased risk of complications in the SNB groups.

With the aging population, the occurrence of knee osteoarthritis is increasing, and TKA is a popular treatment. However, pain control following TKA can be very challenging. Optimal analgesia may shorten hospital stays and result in decreased risks of deep vein thrombosis (DVT) and pulmonary embolism (PE). Furthermore, early rehabilitation exercise contributes to a satisfied sufficient functional recovery. Postoperative pain control is an interesting topic in orthopedic surgery. Multiple perioperative pain management strategies have been implemented following TKA, including femoral nerve block, spinal analgesia, and periarticular or intra-articular injection of anesthetics.

Sciatic nerve block is performed as an adjunct to femoral nerve block in TKA. Several articles have reported its efficiency for pain control compared FNB alone in TKA. Cook et al. [17] suggest that the combined femoral and sciatic provides superior pain management in the early postoperative period after TKA. Pham et al. [18] showed that the combination of continuous femoral and SNB improves analgesia and decreasing opioids consumption and risk of complications.

Quadriceps strength is a major concern following TKA, as quadriceps function is closely associated with postoperative walking and stair climbing ability. The possible etiologies may be muscle strength reduction before operation, patient positioning during operation, long tourniquet times, and inadequate postoperative pain control. Peripheral nerve injury is iatrogenic factor which may cause an increased risk of falls. It has been reported that the rate of peripheral nerve injury is 2.9/10,000 for FNB and 2.4/10,000 for SNB, and the incidence of permanent nerve damage is 1.5/10,000 [19]. Sciatic nerve injury is also a generally known complication after TKA, with an incidence of 1.3 to 2.2% [20, 21]. However, some degree of quadriceps weakness was also observed in LIA group. The data were not sufficient for a meta-analysis; larger sample size of RCTs was needed to reach a conclusion.

LIA was alternative choice to achieve comparable pain control. It was more and more popular for the ease of preform and less motor block. Many kinds of local anesthetics have been applied in TKA. Long-acting local anesthetics including ropivacaine and levobupivacaine are commonly used. In present meta-analysis, all included articles used local ropivacaine for peripheral nerve block whose concentration ranged from 0.2 to 0.5%. Five used ropivacaine for local infiltration anesthesia and one applied levobupivacaine. The present meta-analysis indicated that SNB-combined FNB had an analgesic effect that was superior to that of LIA-combined FNB at 24 and 48 h following TKA. Considering that only six studies were included in present meta-analysis, we did not perform a subgroup analysis for types of anesthetics. Further investigation was necessary.

TKA is usually associated with severe pain in 60% and moderate pain in 30% of patients, especially in the first 48 h, and after postoperative mobilization, pain remains intense [22]. Additional opioids, including oral and patient-controlled analgesia (PCA) administration, were applied as concomitant pain control. Opioid consumption is considered an objective method to measure pain. Opioid-related adverse effects, such as nausea, vomiting, respiratory depression, and pruritus, were reported in previous studies [23, 24]. Besides the side effects described above, drug dependence is also an important issue that should be considered. Minimizing opioid consumption would improve patient satisfaction and expedite mobilization and rehabilitation. The present meta-analysis showed that there was a decreased morphine consumption in the SNB groups compared to LIA groups at postoperative 24 h; however, no significant difference was found between groups regarding the morphine consumption at postoperative 48 h.

Nausea and vomiting are common side effects that are frequently associated with PCA of morphine. Sufficient anesthetic techniques can reduce morphine consumption and subsequently decrease the risk of complications. The present meta-analysis showed that there was a decreased risk of nausea and vomiting in SNB groups compared controls. Considering that only six studies were included in our meta-analysis, we did not perform investigation on dose dependence. Large sample sizes from high-quality RCTs are needed.

There were several potential limitations that should be noted. (1) Only six studied were included in present meta-analysis; although all of them are recently published studies, the sample size is relatively small. We also included non-RCTs; thus, the evidence level would be decreased. (2) Some methodological weakness existed in some included studies which generated potential bias. (3) Functional outcome is an important parameter; due to the insufficiency of relevant data, we fail to perform a meta-analysis; (4) Dose of anesthetics is varied, and concomitant pain management regime differs from each other, which may influence the results of the meta-analysis. (5) Subgroup analysis was not performed due to the small included studies. (6) The duration of follow-up is relatively short which leads to underestimating complications. (7) Publication bias in present meta-analysis may influence the results.

Despite the limitations above, this is the first meta-analysis from recently published studies to assess the efficiency and safety between LIA and SNB when combined with FNB following TKA. Long term of high-quality RCTs were needed to explore the functional outcome of the knees and other adverse effects.

## Conclusion

FNB-combined SNB provides superior pain relief and less morphine consumption within the first 24 h compared FNB-combined LIA in total knee arthroplasty. In addition, there were fewer side effects associated with SNB. Because the sample size and the number of included studies were limited, a multicenter RCT is needed to identify the effects of the two kinds of methods and further work must include range of motion analyses and functional test.

## Abbreviations

LIA: Local infiltration analgesia
RCT: Randomized controlled trials
SNB: Sciatic nerve block
TKA: Total knee arthroplasty
VAS: Visual analogue scale
